# Unilateral renal artery stenosis presented with hyponatremic-hypertensive syndrome – case report and literature review

**DOI:** 10.1186/s12882-019-1246-9

**Published:** 2019-02-21

**Authors:** Jhao-Jhuang Ding, Shih-Hua Lin, Jin-Yao Lai, Tai-Wei Wu, Jing-Long Huang, Hung-Tao Chung, Min-Hua Tseng

**Affiliations:** 1 Division of Nephrology, Department of Pediatrics, Chang Gung Memorial Hospital and Chang Gung University, No 5, Fu-Shing ST., Kwei-Shan, 33305 Taoyuan, Taiwan; 2 Department of Pediatrics, National Defense Medical Center, Tri-Service General Hospital, Taipei, Taiwan; 3 Division of Nephrology, Department of Medicine, National Defense Medical Center, Tri-Service General Hospital, Taipei, Taiwan; 4 Division of Pediatric Surgery, Department of Surgery, Chang Gung Memorial Hospital, Chang Gung University, College of Medicine, Linkou, Taiwan; 5 Department of Pediatrics, Fetal and Neonatal Institute, Division of Neonatology Children’s Hospital Los Angeles, Keck School of Medicine, University of Southern California, Los Angeles, CA USA; 6 Division of Allergy, Asthma and Rheumatology, Department of Pediatrics, Chang Gung Memorial Hospital and Chang Gung University, Taoyuan, Taiwan; 7 Division of Cardiology, Department of Pediatrics, Chang Gung Memorial Hospital and Chang Gung University, Taoyuan, Taiwan

**Keywords:** Hyponatremic-hypertensive syndrome, Renal artery stenosis

## Abstract

**Background:**

Renal artery stenosis is one of the secondary causes of pediatric hypertension. Cases with critical unilateral renal artery stenosis manifesting with the hyponatremic hypertensive syndrome are rare and a comprehensive description of this disorder in the pediatric population is lacking in the literature.

**Case presentation:**

We describe a 4-year-old boy who presented with severe hypertension, profound hyponatremia, hypokalemia, nephrotic range proteinuria, and polyuria. Distinctly, the diagnosis of hyponatremic hypertensive syndrome secondary to unilateral renal artery stenosis was confirmed in light of laboratory and radiographic findings of severe natriuresis, elevated renin, and unilateral small kidney. Two weeks following nephrectomy, there was resolution of hyponatremia, hypokalemia, nephrotic range proteinuria and hypertension.

**Conclusions:**

Findings of hyponatremia, hypokalemia, hypertension, polyuria, and unilateral renal hypoplasia can be attributed to a unifying pathology of unilateral renal artery stenosis.

**Electronic supplementary material:**

The online version of this article (10.1186/s12882-019-1246-9) contains supplementary material, which is available to authorized users.

## Background

Hypertension in children necessitates prompt work-up and diagnosis in order to uncover and appropriately treat secondary causes, such as coarctation of the aorta, renal parenchymal diseases, renal artery stenosis, and endocrine disorders. Concurrent hyponatremia in hypertension is most commonly caused by renin-secreting tumors, renal ischemia, and renal artery stenosis after exclusion of medication-induced hypertension [1]. The central pathomechanism that underlies hyponatremic hypertension syndrome (HHS) is the stimulation and activation of the renin-angiotensin-aldosterone (RAA) axis which consequently trigger hypertension through vasoconstriction as well as fluid and salt retention. In cases of unilateral renal artery stenosis, angiotensin II induces pressure natriuresis of the non-stenotic kidney and hence produces the unique finding of hyponatremia in conjunction with hypertension.

Hyponatremic hypertensive syndrome, a disorder of severe hypertension and hyponatremia, could result from any causes of high renin conditions. The most common etiology in children is unilateral renal artery stenosis. It could present with of conscious disturbance or seizure, polydipsia and polyuria, with the characteristics of extreme hypertension, hyponatremia, hypokalemia, and proteinuria. Due to its insidious course and potential fatality, it warrants careful investigation by an astute physician [2]. Without appropriate treatment, hypertensive encephalopathy, retinopathy, cardiomyopathy, and nephropathy can develop.

Herein, we present a 4-year-old boy with HHS, caused by unilateral renal artery stenosis, featured by hypertension, hyponatremia, polyuria, and polydipsia. After nephrectomy, he achieved full clinical recovery without sequelae.

## Case presentation

**Fig. 1:**
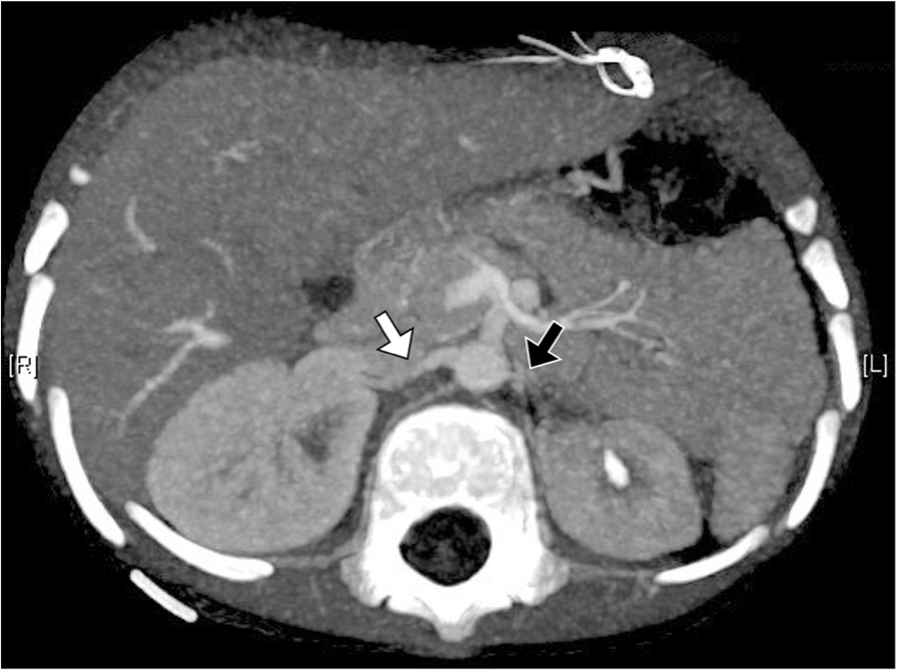
Computed tomographic angiography with maximum intensity projection. The axial view demonstrated the small caliber of the left renal artery (black arrow) with the compensatory change of the contralateral renal artery (white arrow)

A 4-year-old boy, who had no systemic or inherited disease, presented with a 3-week history of intermittent vomiting without diarrhea or abdominal pain. In the past year, he experienced polydipsia and polyuria. Physical examination revealed body weight 17.5 kg (50th percentile), body height 100 cm (15~50th percentile), blood pressure 230/120 mmHg, heart rate 138 /min, and decreased skin turgor. There was no focal neurological deficit, blood pressure discrepancy between upper and lower extremities, palpable mass, nor any appreciation of an abdominal thrill. Laboratory studies revealed serum Na^+^ 124 mmol/L, K^+^ 2.4 mmol/L, Cl^−^ 87 mmol/L, Ca^2+^ 8.5 mg/dL, HCO_3_^−^ 34.5 mEq/L, creatinine 0.41 mg/dL, albumin 3.4 g/dL, IgG 247 mg/dL, and osmolality 290 mOsm/KgH_2_O. Urine analysis was significant for creatinine 11.2 mg/dL, Na^+^ 24 mEq/L, K^+^ 18 mEq/L, Cl^−^ 24 mEq/L, osmolality 232 mOsm/KgH_2_O, RBC 168/μL, FENa 6%, and nephrotic-range proteinuria (55 mg/m^2^/hour). Survey for possible glomerulonephritis demonstrated the absence of anti-streptolysin O, p-ANCA, c-ANCA, ANA, and normal immunoglobulin A, C3, and C4 levels. In addition, work-up for secondary hypertension included: free T4 1.51 (normal range 0.8–2.0 ng/dL), TSH 5.7 (normal range 0.25–5.00 μIU/mL), cortisol 40.18 (normal range 4.3–25 μg/dL), ACTH 9.32 (normal range < 46 pg/mL), renin 1745 (normal range 2–15 ng/L), aldosterone 92.6 (normal range 4–25 ng/dL), and urine vanillylmandelic acid 3.8 (normal range 1.9–9.9 g/day). Renal ultrasonography revealed hyperechoic right kidney (7.6 cm in length) and small left kidney (5.3 cm in length). Due to the presence of hyperreninemic hypertension, natriuretic-hyponatremia, hypokalemia, and nephrotic range proteinuria, HHS was highly suspected. Computed tomography angiography confirmed high-grade renal artery stenosis with hypoplasia of the left kidney (Fig. 1).

In terms of management for this case, we began with volume repletion by normal saline administration. Subsequently, his blood pressure declined from 210/120 mmHg to 180/90 mmHg. Intravenous calcium channel blocker was used to treat his hypertensive emergency, while oral captopril was prescribed for RAA axis blockage after diagnosis of unilateral renal artery stenosis. The systolic blood pressure gradually declined to 150~160 mmHg on the 3rd day. Potassium supplement was infused for his profound hypokalemia and generalized muscle weakness. Due to the severity of left renal artery stenosis, he was not a candidate for angiographic intervention, and decision was made to proceed with left nephrectomy. Overall, electrolyte abnormalities such as hyponatremia and hypokalemia were corrected within 1 week after admission, and resolution of polyuria, polydipsia, proteinuria, and hypertension were achieved 2 weeks after nephrectomy (Additional file 1: Table S1).

## Discussion and conclusions

This 4-year-old boy presented with severe hypertension and volume depletion. Comprehensive examinations excluded the possibility of coarctation of great vessels and renal parenchymal diseases and pointed towards an overactive renin-angiotensin-aldosterone axis. The presentation of natriuretic-hyponatremia, hypokalemia, polyuria, nephrotic range proteinuria, and hyperreninemic-hypertension was highly suggestive of HHS. Aside from HHS, primary neurologic diseases such as intracranial hemorrhage or malignancy may also cause hypertension and hyponatremia, secondary to the increase in intracranial pressure and inappropriate secretion of antidiuretic hormone. However, these diseases usually present with focal neurologic deficits and decreased urine output, which could be distinguished from the polyuria and volume depletion of HHS caused by the renal artery stenosis.

**Fig. 2:**
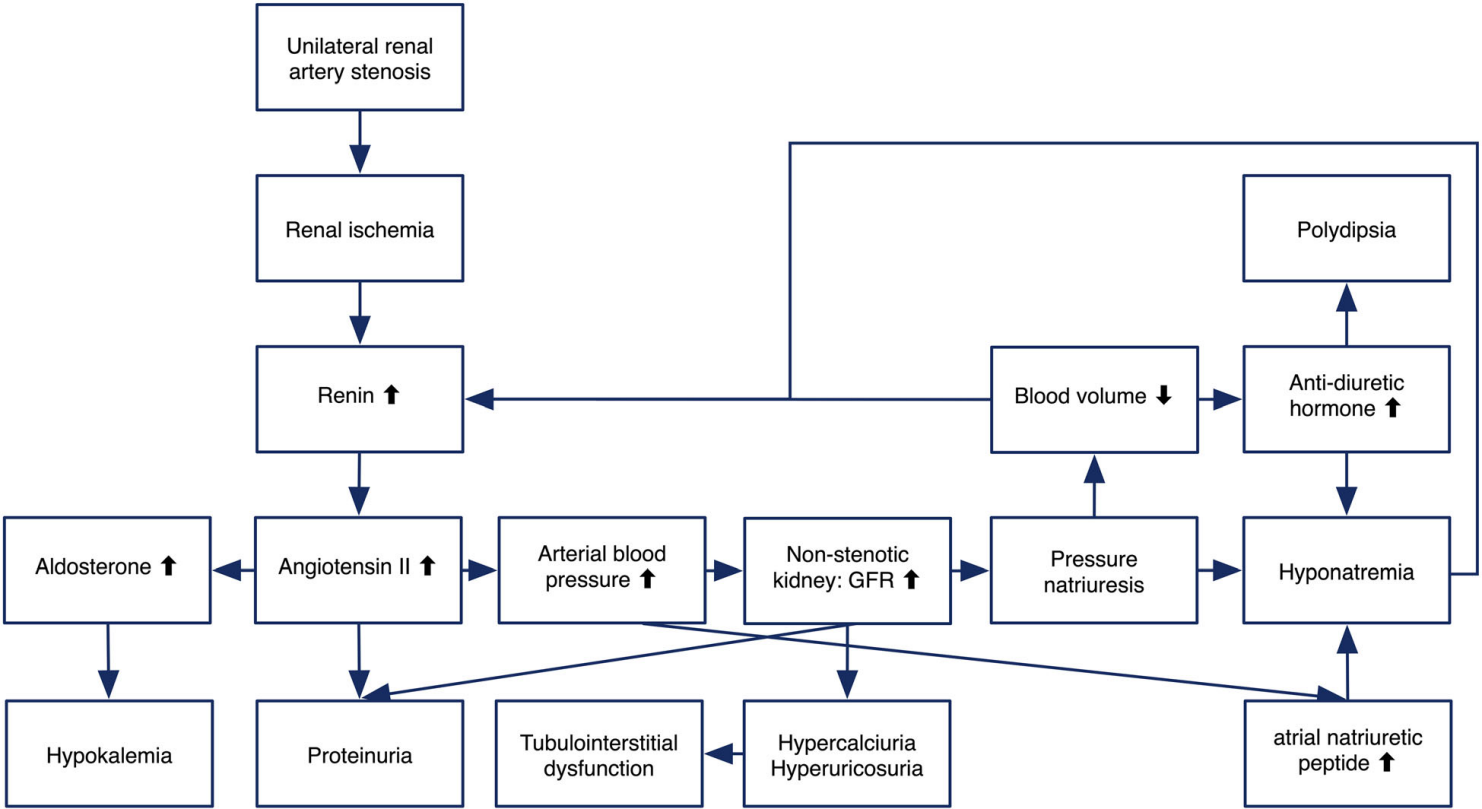
Possible mechanism of hyponatremic-hypertensive syndrome

The main pathogenesis of HHS is renal ischemia, as shown in Fig. 2. Hypertension is induced by stimulation of unremitted renin secretion and subsequent angiotensin II-induced vasoconstriction and secondary hyperaldosteronism. Elevated circulating angiotensin II can cause glomerular hyperfiltration and subsequential pressure natriuresis of the non-stenotic kidney, which results in the clinical presentation of hyponatremia [3]. In addition, volume depletion contributes to the development of hyponatremia by stimulating the secretion of the anti-diuretic hormone. Sodium wasting and volume depletion further stimulates the renin excretion [4]. Hyperaldosteronism, secondary to hyperreninemia and volume depletion, lead to hypokalemia which is one of the leading complications of HHS. Glomerular hyperfiltration of contralateral healthy kidney, deriving from hyperreninemia-induced hypertension, could eventually result in tubulointerstitial injury from the effects of hypercalciuria and hyperuricosuria [5]. Proteinuria in cases of HHS, sometimes in nephrotic range, can result from the glomerular hyperfiltration, proteinuric effect of angiotensin II, and/or consequence of tubulointerstitial injury caused by prolonging hypercalciuria and hyperuricosuria [6].

We conducted a careful search of literature and found a total of 15 reported pediatric cases, as shown in Table 1. The mean age at onset was 4.03 ± 3.38 years with male predominance (11/15). The combination of hypertension, polydipsia, and polyuria are the most common presentations (14/15), followed by hyponatremic seizure (7/15). The mean serum sodium, potassium, and bicarbonate levels were 123.4 ± 5.5 mEq/l, 2.9 ± 0.5 mEq/l, and 28.9 ± 3.5, respectively. Eight of the patients had proteinuria. Excluding the three patients whose renin and aldosterone data was unavailable, almost all patients had hyperreninemia (10/11) and hyperaldosteronism (12/12). The most common extra-renal involvements were neurological (8/15), cardiac (7/15), and retinal (5/15).

**Table 1:** Summary of Clinical Characteristics of Reported Pediatric Cases

Patient [Ref]	Gender/ age	Presentations	BP (mmHg)	Renin (range)	Aldosterone	SNa (mEq/L)	SK (mEq/L)	SHCO3- (mEq/L)	Proteinuria	Organs involvement	Treatment	Outcome
1 [2]	F/ 2y9m	Polydipsia, polyuria Presyncope	215/156	Low	Elevated	129	3	27	700 mg/day	CNS^a^ Kidney	IV *β* blocker, oral *β* blocker, CCB^b^, ACEI^c^ ^d^PTA	Recovery
2 [2]	M/ 2y3m	Polydipsia	142/92	NA	Elevated	122	3.9	25.9	3200 mg/day	Heart Kidney	IV *β* blocker, oral *β* blocker, CCB, ACEI PTA	Recovery
3 [2]	M/ 2y	Polydipsia, polyuria Restlessness	220/150	Elevated	Elevated	125	3.2	27.2	5300 mg/day	Heart Kidney	IV *β* blocker, oral *β* blocker, CCB, ACEI PTA	Recovery
5 [9]	M/ 1y6m	Seizure, hemorrhagic and ischemic stroke	210/160	172 ng/ml/min (3~11)	91 ng/dl (4~16)	120	2.1	NA^e^	NA	CNS Heart Kidney	Nitroprusside, IV *β* blocker, oral CCB Aorto-renal bypass	Hypertension
4 [14]	F/ 1y3m	Polyuria, polydipsia	190/120	24 ng/ml/hr. (1~4.5)	8 nmol/l (0.1~0.8)	122	2.4	29.5	1800 mg/day	Heart Kidney	ACEI, *β* blocker, CCB, spironolactone PTA	Recovery
6 [15]	M/ 7y	Polydipsia, polyuria	210/120	NA	NA	114	2.4	NA	NA	Retina Kidney	CCB, *α*1 blocker Nephrectomy	Recovery
7 [16]	M/ 2y9m	Polydipsia, polyuria Seizure	160/120	80.44 ng/ml/hr. (0.2~2.8)	6861 pg/ml (10~160)	118	1.9	NA	NA	CNS Kidney	CCB, desmopressin ACEI Nephrectomy	Recovery
8 [16]	F/ 1y4m	Polyuria, polydipsia	140/90	NA	NA	131	2.6	NA	NA	Kidney	CCB ACEI Nephrectomy	Hypertension
9 [17]	M/ 9y	Polyuria, polydipsia Seizure	156/120	NA	NA	124	3.2	34	NA	CNS Retina Kidney	Nitroprusside ACEI, CCB PTA with stenting	Hypertension
10 [18]	M/ 1y7m	Polyuria Polydipsia Seizure	248/150	137 ng/ml/min (3~11)	743 ng/dl (7~93)	128	3.2	24	NA	CNS Heart Kidney	Nitroprusside, ACEI PTA with stent	Hypertension
11 [19]	M/ 5y	Seizure	236/132	21.06 ng/ml/hr. (1.3~3.9)	1172 ng/dl (1~16)	112	3.2	33.4	^f^UP/UCr 6.84	CNS Kidney	*β* blocker, CCB, hydralazine	Proteinuria Hypertension
12 [19]	M/ 8y	Polydipsia, polyuria Seizure	184/110	32.8 ng/ml/hr. (1.3~3.9)	1436 ng/dl (1~16)	127	3.1	27.2	UP/UCr 3.91	CNS Retina Kidney	*β* blocker, CCB, hydralazine PTA	Recovery
13 [19]	M/ 12y	Polydipsia, polyuria Seizure	244/166	25.04 ng/ml/hr. (1.3~3.9)	1358 ng/dl (1~16)	126	3.2	32.2	UP/UCr 4.36	CNS Retina Kidney	*β* blocker, CCB, hydralazine PTA with stenting	Hypertension
14 [20]	M/ 2y	Polydipsia, polyuria	NA	2537 ng/dl	31.6 ng/dl	124	2.8	NA	1230 mg/day	Heart Kidney	Hydralazine Angioplasty with patch	Recovery
15 [20]	F/ 2y	Polydipsia, polyuria	NA	76.5 ng/dl	48.1 ng/dl	128	2.7	NA	2400 mg/day	Heart Retina Kidney	CCB, *β* blockers, hydralazine Angioplasty with patch	Recovery
Our case	M/ 4y	Polyuria, polydipsia	230/120	174.5 ng/dl	9.26 ng/dl	124	2.4	34.5	55 mg/m^2^/hr	Kidney	IV CCB, Oral ACEI Nephrectomy	Recovery

^a^CNS, central nervous system; ^b^CCB, calcium channel blocker; ^c^ACEI, angiotensin-converting enzyme inhibitor; ^d^PTA, percutaneous transluminal angioplasty; ^e^NA, not available; ^f^urine protein-to-creatinine ratio (mg/dl/ mg/dl)

The mainstay of treatment for renal artery stenosis-associated HHS lies in the restoration of intravascular volume, prevention of acute insult of hypertensive crisis and correction of underlying renal arterial stenosis. Volume depletion needs to be corrected first to improve systemic blood flow and prevent further injury resulting from renal ischemia [7]. After volume repletion, the prompt decline of blood pressure could be achieved by intravenous calcium channel blocker, which has been suggested to be the first line drug for severe hypertension with acute kidney injury [8]. For cases with HHS, angiotensin-converting enzyme inhibitor and angiotensin II receptor blocker should be introduced to mitigate the over-activation of the RAA system [9]. However, the use of diuretics is not recommended due to the potential deleterious effects of fluid and sodium wasting which could further activate the RAA system [10]. Lastly, correction of renal artery stenosis can be achieved surgically by percutaneous renal angioplasty, renal artery reconstruction, or nephrectomy. As shown in Table 1, all patients received anti-hypertensive agents as the first line therapy. Eleven and three cases underwent angioplasty and unilateral nephrectomy, respectively. It is important to note that HHS caused by renal artery stenosis does not always result in a favorable outcome, as five patients had residual hypertension despite aggressive treatment. Several causes of residual hypertension in cases with HHS have been proposed. A longitudinal pediatric study stated that over 40% of renal artery angioplasty would develop restenosis [11]. Also, chronic kidney disease caused by prolonging tissue hypoxia and consequence of proteinuria could lead to hypertension despite restoration of renal blood flow [12]. Finally, uncontrolled hypertension itself could cause irreversible remodeling of vascular endothelium, resulting in permanent hypertension [13].

In conclusion, HHS caused by unilateral renal artery stenosis is a potentially curable and reversible disease when promptly diagnosed and appropriate treatment is implemented. Hyperreninemic hypertension, natriuretic hyponatremia, nephrotic range proteinuria, and unilateral renal hypoplasia are clinical clues that aid in uncovering the diagnosis.

## Additional file

Additional file 1: **Table S1.** Clinical and laboratory characteristics before and after treatment. (DOC 32 kb)

## Abbreviation

HHS: Hyponatremic hypertension syndrome
