# Supplementary material for: Unilateral renal artery stenosis presented with hyponatremic-hypertensive syndrome – case report and literature review
Source: BMC Nephrol. 2019 Feb 21;20:64. doi: 10.1186/s12882-019-1246-9 (PMC6385391; doi:10.1186/s12882-019-1246-9)
Supplement: Supplementary file 1 — Table S1. Clinical and laboratory characteristics before and after treatment. (DOC 32 kb) [file 12882_2019_1246_MOESM1_ESM.doc]

**Table S1**. **Clinical and laboratory characteristics before and after treatment**

|  | At presentation | After treatment |
| --- | --- | --- |
| Blood pressure (mmHg) | 233/150 | 98/53 |
| Urine output (ml/kg/hour) | 67.5 | 2.4 |
| Biochemical data |  |  |
| Serum Na^+^ (133-143 mmol/l) | 129 | 140 |
| Serum K^+^ (3.5-5.5 mmol/l) | 2.4 | 4.2 |
| Serum Cl^-^ (92-102 mmol/l) | 85 | 107 |
| Serum HCO3^-^ (22-26 mmol/l) | 34.6 | 23.2 |
| Plasma Renin (ng/L) | 1745 | 17 |
| Plasma Aldosterone (ng/dL) | 92.6 | 5.2 |
| Urine protein (mg/m^2^/hour) | 55 | 2.6 |
